# Severe hypocalcaemia and hypomagnesemia presenting with severe neurologic and gastro-intestinal symptoms: a case report and review of literature

**DOI:** 10.1007/s43678-021-00089-9

**Published:** 2021-02-05

**Authors:** F. J. de Boer, I. van Ieperen, H. E. Boersma, H. R. Bouma

**Affiliations:** 1 Department of Internal Medicine, University Medical Center Groningen, University of Groningen, EB40, P.O. Box 30.001, 9700 RB Groningen, the Netherlands; 2 Department Clinical Pharmacy and Pharmacology, University Medical Center Groningen, University of Groningen, Groningen, the Netherlands

**Keywords:** Delirium, Calcium, Magnesium, Drug interaction

## Case presentation

A 65-year old male patient presented to the ED with general weakness, vomiting and diffuse abdominal pain for several days. There was a history of pulmonary embolism and recent repeated autologous stem cell transplantation due to recurrent multiple myeloma 2 months ago. His medication included rivaroxaban, esomeprazole and fluconazole. The latter was prescribed during the latest visit to the outpatient clinic for an oropharyngeal infection that was presumably caused by *Candida* spp. On arrival conversation was not possible. Medical history taken from his wife revealed that the epigastric pain started 5 days ago with subsequent vomiting for 2 days. Fever, chills or abnormal stools were not reported. On examination patient appeared moderately ill, was disoriented and unable to communicate. Further physical examination revealed normal heart and lung sounds, active bowel sounds and mild diffuse abdominal tenderness to deep palpation without involuntary guarding or rebound tenderness. Neurological examination revealed a tremor without other neurological deficits apart from aphasia and confusion. Electrocardiography (ECG) showed a sinus rhythm (76 bpm) with a right bundle branch block (RBBB) and a prolonged QT-interval (QTc 511 ms). Our differential diagnosis was delirium due to ileus or abdominal sepsis, primary cerebral pathology (including intracerebral hemorrhage, tumor or ischemia) or electrolyte disorder (e.g. hypercalcemia, hyponatremia). Point-of-care blood gas analysis revealed a metabolic alkalosis (pH 7.59, pCO2 3.4 kPa [25.51 mmHg], HCO3 24 mmol/L), hypocalcaemia (1.63 mmol/L; corrected for albumin; ionized 0.77 mmol/L) and hypokalemia (2.9 mmol/L, while additional blood analysis revealed a hypomagnesemia (0.13 mmol/L). A computed tomography (CT) scan of the brain was made that showed no abnormalities. Based on a working diagnosis of severe hypocalcaemia with major neurologic and gastro-intestinal symptoms, we administered one ampule of calcium gluconate (10 ml, which contains 1 g [2.25 mmol] of calcium) intravenously over 10 min. His aphasia, confusion and nausea recovered within minutes after calcium supplementation and the QT-interval normalized. Additional laboratory analysis revealed no increase in inflammatory parameters. Urinary magnesium excretion was low (0.56 mmol/L) and potassium excretion was high (74 mmol/L). We concluded severe hypomagnesemia with secondary hypocalcaemia and hypokalemia in our patient to be due to a drug-drug interaction between the fluconazole that was initiated 10 days prior to presentation and esomeprazole, leading to increased plasma levels of the latter. Previous measurements of magnesium, potassium and calcium, as was performed 1 month before presentation, had been within the normal range. Profuse vomiting likely aggravated the electrolyte disturbances. The patient was admitted to the ward for further supplementation of calcium, magnesium and potassium, while esomeprazole was discontinued. He could be discharged in good condition 3 days later.

## Discussion

Calcium is critical for contraction of smooth, skeletal and cardiac muscles and nerve conduction. The hallmark of acute, severe hypocalcaemia is neuromuscular irritability: hyperexcitability (tremor, tetany and convulsions) or weakness, and a delayed cardiac repolarization that may be noted as a prolongation of the QT-interval [1]. Neurologic symptoms like delirium, aphasia or depression can also occur [2]. Diagnostic clues that may be found on physical examination are Trousseau’s and Chvostek’s sign and carpopedal spasm. Symptoms generally occur when serum calcium is below 1.9 mmol/L. The initial treatment of hypocalcaemia consists of intravenous supplementation with calcium gluconate in symptomatic patients or when serum calcium is less than 1.9 mmol/L. This can be continued as continuous infusion or oral supplementation with control of calcium concentrations [1]. Causes of hypocalcaemia are vitamin D deficiency, (pseudo)hypoparathyroidism, gastro-intestinal loss, medication (i.e. bisphosphonates, phenytoin, and cinacalcet), critical illness (i.e. sepsis, acute pancreatitis) and renal insufficiency [1, 2]. In our case, hypocalcaemia likely resulted from the severe hypomagnesemia mediated by decreased parathyroid hormone (PTH) secretion or PTH resistance. Magnesium plays a central role in PTH secretion and end-organ effects of PTH. Hypomagnesemia may be caused by reduced intake, gastro-intestinal loss, proton pump inhibitor use, increased renal tubular flow (i.e. osmotic diuresis, diuretics, antibiotics, cisplatin) or acute tubular necrosis and endocrine causes (i.e. hypercalcemia, hyperthyroidism). Symptoms of hypomagnesemia typically occur at serum magnesium levels below 0.5 mmol/L and are very similar to symptoms of hypocalcaemia, given the critical role of magnesium in the transport of calcium over the cell membrane. Neuromuscular irritability (i.e. weakness, tremor and convulsions) and a prolonged QT-interval with flattening of T waves on the ECG can occur, but also gastro-intestinal symptoms as abdominal pain and vomiting [3, 4]. Additionally, hypomagnesemia can lead to hypokalemia as magnesium is required for renal potassium reabsorption. The metabolic effects of magnesium may render hypocalcaemia and hypokalemia resistant to treatment if there is concurrent hypomagnesemia [5]. Hence, it is important to also correct hypomagnesaemia in patients with hypocalcemia or hypokalemia with intravenous magnesium sulfate or oral magnesium gluconate in symptomatic patients or when serum magnesium is less than 0.5 mmol/L. Importantly, when severe hypomagnesemia is suspected, specifically when the QT-interval is prolonged, empiric suppletion should be considered while the investigations are ongoing.

Proton pump inhibitor use is a common cause of hypomagnesemia, presumably due to impaired intestinal absorption, caused by inhibition of selective channels (TRPM-6) in the duodenum by the proton pump inhibitor. Urinary magnesium excretion is low [4, 6]. Our patient used esomeprazole in a relatively high dosage (40 mg/day) and recently started fluconazole treatment. The combination of these drugs caused a drug-drug interaction leading to a significant rise in the plasma concentration of esomeprazole. Esomeprazole, the *S*-isomer of omeprazole, is metabolized by CYP2C19 (73%) and CYP3A4 (27%) and has a lower clearance than *R*-omeprazole or omeprazole. Consequently, administration of esomeprazole leads to higher plasma levels as compared to administration of the same dose of omeprazole [7, 8]. Moreover, co-administration of fluconazole, which is a CYP3A4/CYP2C19 inhibitor can lead to more than doubling of the maximum plasma concentration (*C*_max_) of (es)omeprazole [9]. Thus, co-administration of fluconazole likely led to a esomeprazole intoxication with serious electrolyte disturbance due to a drug-drug interaction that was likely not recognized by the prescriber.

## Conclusion

We present a case of a severe hypocalcaemia, hypomagnesemia and hypokalemia due to a drug-drug interaction of esomeprazole and fluconazole, presenting with gastro-intestinal and neurologic symptoms. This case demonstrates several important learning points. First, severe global deterioration in neurologic functioning, mimicking a delirium or cerebrovascular accident, can be the presenting symptoms of hypocalcemia and hypomagnesemia. Second, unrecognized drug–drug interactions between commonly prescribed drugs can lead to potentially life-threatening electrolyte disturbances. Among these commonly prescribed drugs are proton pump inhibitors of which it is important to realize that these drugs can lead to hypokalemia, hypocalcemia and hypomagnesemia even in the therapeutic range. As drug–drug interactions between seemingly benign drugs can result in potentially significant outcomes, it is important to safeguard this using automated interaction warnings as commonly embedded in electronic prescribing systems and consult the hospital pharmacist when in doubt. Changing the specific drug prescribed, adapting the dose or monitoring electrolytes can be indicated to reduce the risk of a serious adverse event. Third, having a point-of-care blood gas analyzer in the ED allows prompt recognition and treatment of electrolyte disturbances. The initial treatment of symptomatic hypocalcemia consists of intravenous supplementation with calcium gluconate, which should lead to prompt restoration of symptoms.

## Consent and reporting

Informed consent was obtained from the patient. Manuscript was prepared in accordance with CARE guidelines for case reports (Gagnier et al. [10]).
